# Our Summer at Maplewood. Chapter X, Odds and Ends

**Published:** 1875-11

**Authors:** 


### CHAPTER X.

### ODDS AND ENDS.

"Egg-Plant in early August! I thought it never came till September?" interrogatively remarked Cousin Emmeline, as we seated ourselves at table.

"This is a new variety," I replied, as I passed her a slice of the fried vegetable. "I wish your opinion as to whether it is equal in quality to that which comes later in the season."

There was a merry twinkle in Alice's eye, which her mother did not notice, as she answered,—

"Why, this is delicious! I never ate finer egg-plant. Indeed, I think this has a delicacy of flavor I never before observed in the vegetable. Have you some new process of cooking it, by which you extract that peculiar flavor which renders egg-plant obnoxious to many persons?"

"No," I said, giving Alice a silencing glance. "This was fried in the same manner in which I usually fry egg-plant. Cut in slices a quarter of an inch in thickness, rubbed lightly with salt, and piled one slice upon another that some of the water might be drawn from the plant, it was allowed to stand for half an hour. The slices were then wiped dry, dusted very lightly with pepper, and freely with flour, and fried until thoroughly done, and nicely brown, in a small quantity of fat. For frying nearly everything of this sort, a thick cast-iron pan or spider is much better than one made of lighter metal. Equal proportions of lard and butter I find better for frying than either lard or butter alone. A small quantity should be first placed in the pan or spider, and, when hot, the slices of vegetable should be laid in. When nicely browned they should be turned, and more of the butter and lard added. Egg-plant will soak a great deal of fat, if permitted to do so. I therefore put in at first only enough to fry one side; and add sparingly, from time to time, as is necessary. It is well to have a cup of this butter and lard mixture at hand, melted and hot, so that you can put it in the pan with a spoon, when required. To prevent the slices soaking fat, some cooks coat them with egg and bread-crumbs; but I can't see that anything is gained by the operation, as the coating absorbs a good deal of oil, which is eaten with the egg-plant; and when cooked in this way it is liable to be underdone, as it requires much more cooking with than without the coating of crumbs. I also think it far more palatable without the crumbs; but as tastes differ, there is no harm in trying both methods."

"I am exceedingly fond of egg-plant fried according to your instructions, without egg and crumbs," observed Cousin Emmeline, "and think it one of the best of breakfast-dishes. With me it takes the place of meat. May I trouble you for another slice?"

"Give ma fried squash this time, Cousin Kate," said Alice, adding, in a mock tone of pity, "Poor mamma! to be victimized in this way, and made to praise fried squash so enthusiastically, all for the sake of Cousin Kate's horrid cook-book, is too bad! It is positively cruel, I declare!"

"Fried squash!" interrupted Emmeline. "What do you mean?"

"Why, simply this," I explained. "That summer squash, or cymlin, when fried in the same manner as egg-plant, is a good substitute—"

"And not always detected as a substitute," interrupted Alice, mischievously. "In fact, is sometimes mistaken for the genuine article by those very fond of egg-plant."

"Which means, I suppose," said Emmeline, "that this delicious dish I have been eating and praising, is fried squash,—something I never heard of before."

"Exactly," I rejoined; "and by many preferred to egg-plant, on account of being a less hearty food, and having a more delicate flavor. Tomatoes, not over-ripe, cut in thick slices, and fried in the same manner, are very nice. The tomato should be sliced, seasoned, dusted with flour, and fried immediately. The skin should be left on, to prevent the slices from coming to pieces."

"Kate," said Emmeline, "you certainly excel as a fryer,—or, should I say fryist, or whatever the proper term may be? From fried chicken down to fried squash, all your fries are admirable; and if your methods were generally adopted, such a revolution would take place in that sadly abused mode of preparing food that Dr. Trall's dictum of "frying being a very objectionable method of cooking," would lose its force, and the frying-pan would again come into as general use as in the days of our grandmothers. This would comport more completely with Dr. Hall's less radical but more reasonable theory of "all good things in their proper places." Oysters fried after your recipe I always forgot to praise, in my eagerness to enjoy."

"And yet, Emmeline, I have in several instances given my exact method of frying oysters to friends, and when the black, greasy things, came upon the table, although I could not recognize them as the legitimate result of my method at all, the hostess invariably insisted that my directions were followed to the minutest particular. This is a somewhat discouraging experience for one contemplating writing a cook-book. Nevertheless, I suppose I shall go on telling people how to cook, and hopefully look to the future for better results.

"For frying oysters, I like cracker-crumbs rolled very fine and sifted, so that you have almost cracker-flour. The oysters should be taken singly, and well rinsed in their own liquor, or in cold water, so that no particle of shell shall adhere to them, and laid upon a sieve or folded napkin, to drain. While the oysters are draining, season the cracker-crumbs with salt and Cayenne pepper. Mix the seasoning very thoroughly with the cracker-dust; dip each oyster in well beaten egg, and roll it freely in the cracker. Lay it on a plate, or board, convenient to handle. Prepare in this way all the oysters before you begin to fry. A thick dripping-pan or deep griddle answers well for frying them in. When everything is ready, place in the pan an ounce or two of lard. The exact quantity required depends upon the size of the pan and the number of oysters. When the lard is boiling hot, add to it an equal quantity of butter. As soon as the butter is melted and mixed with the lard, fill the pan with oysters, laying them close together. Let the heat be enough to fry quickly, but not so great as to burn. When brown on one side, turn the oysters; and when nicely brown on both sides, lift to a heated platter, and serve immediately. Fried oysters, chicken, potatoes, &c., are supposed to be nicer served on a napkin.

"It is well to let the lard get hot in the pan before the butter is added, as the butter is not so likely to scorch as when put in the pan at first. Place enough of the mixture in the pan before you begin frying, to fry the oysters on both sides; but if a second panful is to be fried, the pan must be washed, and all the burnt grease removed, or the oysters will be badly damaged by it. When properly cooked, the oyster comes to the table of a light brown color, thinly coated all over with crumbs,—not warm, greasy, and leathery, but hot and juicy, and tasting precisely like—a fried oyster!

"For scalloped oysters, prepare the crumbs by drying bread very thoroughly in a cool oven. Broken pieces and crusts will answer as well as whole slices, if the bread is of good quality and the crusts not brown. Several hours will be required to dry the bread sufficiently; and it will not be injured if lightly browned in the process. When dried, roll it very fine, rejecting whatever will not crush into fine crumbs. Season the crumbs well with salt, pepper, and butter, rubbing all between the hands until thoroughly mixed. Rinse and drain the oysters the same as for frying. The dish in which scalloped oysters are cooked should be shallow,—holding not more than three or four layers of oysters and crumbs. Scatter a thin layer of crumbs over the bottom of the dish, and place a layer of oysters upon them. Over these scatter a thin layer of crumbs, and lay in more oysters. So fill the dish, covering each layer of oysters with a layer of crumbs. Over the top place the crumbs thickly, so as to form a coating or crust, which will protect the oysters from exposure. It requires three quarters of an hour to cook scalloped oysters three or four layers deep. The oven should be hotter than for bread—hot enough to cook as quickly as possible without burning the crumbs. The dish should be of a rich brown all over the top, before it is moved from the oven; and when served, the oysters should be plump and juicy. The juice, however, should be found inside the oysters,—while on the outside they should be so dry as to roll and tumble about on the plate. They should not be wet, sticky, and packed together; nor shrunken, leathery, and sloppy, from too much moisture, and from slow cooking or over-cooking. Oysters—whether stewed, fried, scalloped, or broiled,—should never be cooked long enough to collapse and become tough and juiceless."

"Kate, in the matter of food, my sympathies are with country people," observed Emmeline, as we rose from the table. "They usually cook their food in such a careless, slovenly manner, and have so little variety in it, that but for their active out-door lives they would certainly lose all appetite, and die of starvation. It is hard to imagine how people manage to survive, who, day after day, for years, drink the same sloppy coffee, and eat the same sour, heavy bread, and badly cooked meat and vegetables which one is treated to at the average country house! But the worst of it all is that they worry through life on such fare, and never realize the fact that it is possible to prepare food in any other or better way. Your cook-book will give them useful hints on preparing frizzled beef, codfish, &c., but can't you also suggest some improvement in hashes and the warmed-up meats and potatoes which are so often found upon their breakfast-tables? And, by the way, don't forget fish-balls. There is such a vast difference between a perfect fish-ball and a fish-ball as ordinarily made and cooked, that I think the subject deserves special mention."

"Yes," I replied, "the difference between an ordinary and a perfect fish-ball, is as marked and appreciable as between any other properly and improperly prepared article of food. To make perfect fish-balls requires care and attention. Salt fish should be soaked for several hours in cold water, then boiled slowly until very tender. The potatoes should be well boiled, dry, and mealy. Take measure for measure of fish, picked clean from skin and bones, and potatoes. Add a fresh egg, a small piece of butter, Cayenne pepper, and a little sweet milk. Work these ingredients so perfectly together that the fish and potato are indivisible as are two drops of water run together. In this thorough mixing consists the chief art of making codfish-balls. Roll this mixture into round balls between the hands, dust with flour, and fry in boiling lard. If the balls crack and come to pieces in the lard, it is because the lard is not hot enough or the balls are too soft. These round balls, fried in a quantity of boiling lard, will be found far superior, for most tastes, to flat cakes made of the same fish and fried in a small amount of grease. When nicely brown, the ball should be lifted from the lard in a wire skimmer, dried on a napkin, and served hot. When boiled fish, with drawn butter, has been served for dinner, what is left can be made into balls, and the gravy used instead of butter and milk. Delicious balls can also be made of fresh fish, either baked or boiled, and a proportion of light bread soaked in sweet milk, may be used in place of potato.

"All cold fresh meats, when properly hashed and served, make nice, palatable breakfast-dishes. In preparing these dishes great care should be taken to throw out all gristle, tough skin, and dry, chippy portions. The meat should then be hashed very fine, and mixed either with hashed potato or bread crumbed and soaked in sweet milk. Ordinary hash, made of equal proportions of corned beef and potato, can be much improved and made really delicious, by adding bread-crumbs soaked in milk, or sweet milk or cream without the crumbs, and working it well with the hand, then forming it into rolls, and browning in the oven. Or, it may be placed in a well-buttered bread-pan of medium size, and put in a hot oven until it is brown on the bottom and sides, and perfectly heated. When ready to serve, turn it from the pan on a heated platter. Very nice hashed meat-balls or cakes are made by mixing with hashed meat bread crumbed and soaked in sweet milk and a fresh egg. Season to taste with salt and pepper and sweet herbs, if liked. If the meat is mostly lean, sweet cream or a little butter must be added. Make into round cakes, and fry quickly in a small quantity of hot fat. When brown on both sides, serve hot. For frying all these meat-and-potato cakes, the fat fried out of salt pork is much nicer than fresh lard.

"'Scrapple,' made in the following manner, is both palatable and nutritious. Instead of pork, which is generally used for making scrapple, take a piece of beef, or a beef-bone with meat on it, and boil slowly till very tender. Strain the liquor into an earthen bowl, and set aside. Separate the bone from the meat, and, when cold, hash the meat very fine and put it in a kettle with the liquor and half of the fat that has risen on it. Season with salt and pepper; and, when it boils, thicken with corn-meal. Stir in the meal as in making mush or hasty pudding. When thick enough, or as thick as mush, pour into a pan to cool; and, when cold, cut in slices and fry in a skillet or on a hot griddle. In cool weather scrapple will keep several days, and is a convenient breakfast-dish.

"Hashed potato warmed, I think much nicer than that which is coarsely sliced. This is my method of preparing it: Place a small piece of butter in the kettle or pan to be used for warming, and, when melted, add milk or thin cream (which is better), with salt and pepper to taste; and, lastly, add the hashed potato. Cover closely, and set where it will heat slowly. The milk should boil up through and over the potato, and have time to soak into it pretty thoroughly. It should be stirred very little; and, when served, milk should be visible. Hashed potato warmed in this manner is very nice with beefsteak, ham, or cold meat."

---

One afternoon Cousin Emmeline and I took a long walk to the village post-office, and, on our way home, sat down to rest under the grand old oak that stands, like a faithful sentinel, at the entrance to Maplewood. Absorbed with my own meditations, I gazed in silence at the beautiful landscape, until my attention was arrested by an impatient exclamation from Emmeline, who, in looking over the village paper, which was among our mail-matter, had been irritated by the following paragraph:—

"PERSONAL.—The readers of the *Democrat* will be glad to learn that our reporter, after thoroughly investigating the affair, has come to the conclusion that there are no 'spooks' in or about the Douglas mansion; and that the story of ghosts and goblins having been seen and heard there, originated with a nervous old lady, who one evening happened to see some fairy-like garments ornamenting a clothes-line in the garden, and permitted her imagination to get the better of her judgment. The facts in the case, as far as our reporter has been able to gather them, are simply these: Three lady friends of the Douglas family, who are said to belong to the class known as 'strong-minded women,' have been spending the summer at Maplewood, and doing the principal part of their own housework for the sake of privacy and seclusion. Rumor has it that they have been making experiments in cookery, &c., and intend getting up a book, in which instructions will be given for preparing food in a practical manner. We hope this latter report may prove true; for the cook-book has yet to be written in which concise directions are given for plain cooking; and improvements in the cooking of the average American housewife are certainly greatly to be desired. It is said the Douglases intend coming home shortly; and, it is also whispered among gossips that one of the present occupants of their mansion is a beautiful young lady, who, travelling with her mother in Switzerland last summer, fell in with the Douglas party; that a sudden attachment sprung up between the young people; and that, ere many months, a new mistress will occupy the old manse."

"Kate," she said, after I had finished reading the obnoxious article, "I'd much rather Alice wouldn't see that. I think it would annoy and vex her exceedingly. And if it did not do that, it might start her on a new train of thought more objectionable than that she is now pursuing. Do you know the child's behavior astonishes me. Educated in a convent, and always accustomed to the strict formalities of the church, one would naturally suppose she would have been shocked at the doubts and sentiments expressed by Gerald in his wild ravings about his dead wife. But the other day, when I said something of the sort to her, she simply answered, 'Why so, mamma? I've had very similar thoughts myself. Quite as wicked, I dare say; but I never could have given them such beautiful expression.' I confess to you, Kate, I'm becoming seriously annoyed. I don't like this hero-worship she is evidently indulging in. Why, the child is only twenty; and I should prefer to have her keep clear of love's entangling alliances until she is twenty-five, at least."

"True," I replied, "but you know 'Satan finds some mischief still for idle hands to do.' And that which is true of hands is equally true of brains and hearts. If you don't wish Alice to think about Gerald, and fall in love before her time, give her something else to think about, and something to do. Now, if you were to become heartily enlisted in this school project of mine, who knows but she might join in the work also, and become so enthusiastically interested in it, that love would be put away from her thoughts forever."

"No,—not forever, I hope," said Emmeline; "that would be too long. But what is this school project you speak of? Do you seriously contemplate taking charge of a school for girls?"

"Seriously, I do," was my reply. "Heretofore, as you know, I have done a good deal of talking; hereafter, I propose doing some honest work. It seems to me of vital importance that girls should be thoroughly instructed in the domestic arts. Of these, I consider the culinary department the most important. But a practical knowledge of all of them is essential to the comfort and well-being of a household. Glance over the homes to which you have free entrance. Look at the slack, slovenly way, in which most of them are ordered. Housework is considered drudgery of the meanest sort, and is pushed aside whenever possible; or, if not, is done in a fretful, complaining spirit. Servants, as a class, are poorly educated in their department; but the average servant, who professes to do general housework, is fully up to the capacity of the average mistress. Where a servant habitually makes poor bread, serves badly cooked food, or does her work generally in a sleazy, slipshod manner, you will find the mistress powerless to correct the evils, because of her ignorance of the proper methods. Here, emphatically, 'knowledge is power'; and Bridget misrules the household, because her ignorance is less dense than the ignorance of the mistress. Now, in this school of mine the girls are to be taught home duties and domestic work; and, at the same time, to be inspired with a love of them."

"How?" asked Emmeline, all attention.

"Insensibly, in a thousand ways," I continued. "In the first place, the school is to be a home; and is to be as charming and attractive as it can be made. Everything about it will be convenient, comfortable, cosy. The housekeeping department will be administered with great care; and the girls will see, every day and all the time, housework properly performed. The table will be abundantly supplied with the best food, prepared in the most careful manner, after the most approved methods. In short, my ideas are, that study, under proper conditions, is conducive to thorough health; that the highest mental culture is productive of the most perfect physical development; and that young women, while being qualified for the duties of life, can be developed mentally and physically, and inspired with a genuine abiding love of domestic as well as intellectual pursuits. Emmeline, you may consider me an enthusiast; but I expect to live long enough to see these theories of mine, if you choose to call them such, demonstrated as facts at this Home-School, or home and school combined."

"But, Kate, after you get through with cooking, I don't see what will remain for your girls to learn in the housekeeping department. Every woman of sense knows how to do general housework, — to wash and iron, make beds, sweep, &c."

"No, Emmeline; I think very few women know the best ways of doing any of these things. I expect to deliver a lecture, at least once a week, to the girls in the domestic department, on such subjects as — how to make fires, with the special treatment of coal fires; how to manage drafts and dampers; how to keep a coal fire at a low ebb, yet ready to be forced to a quick heat in a few minutes; how to sweep with a common broom without raising a dust; how to make and use baking and ironing holders; how to make beds; and so on, indefinitely. And in this connection let me give you a little story, just in point. One day, when travelling in the West, I fell into conversation with a gentleman who occupied the seat next to mine in the cars. Our talk was rambling and desultory. He, I learned, was president of an Eastern college, who had been spending his vacation in Minnesota. He had a lot of guns and fishing-tackle with him, and had evidently been having a good time. 'What a perfect place,' he said, 'Minnesota is for a summer vacation. The climate is delightful during July and August. How pure and bracing the atmosphere is on the hottest days. And the nights are so delightfully cool that no one thinks of sleeping without blankets,—double blankets, at that.' And here an expression of pain clouded his genial face. After a moment's hesitation, however, he continued, 'I wish something could be done—but I suppose nothing can—to cure chambermaids of a stupid habit they have, which has caused me more serious annoyance during my life, I think, than any other one thing. I've been tempted several times to write a newspaper article upon the subject, and call attention to it as a public and general nuisance, but I have never done so. You may look upon it as a trifle; but I assure you I found it a very serious matter the other night at a hotel in St. Paul. I had been out tramping all day, and came in very tired. The evening was cool; and when I crawled under the blankets at bedtime, I had no presentiment of coming evil. But after a sound sleep, I awoke, sweltering, in torments, loaded with blankets. Upon a careful examination I discovered I had been sleeping under two heavy blankets in August. I attempted to turn one away, but found they would not be separated. Both would go, or both would stay. It was dark, and I had no matches; so I tugged, and labored, and toiled; but my efforts to separate the blankets were ineffectual. Eventually I discovered the cause of failure: the stupid chambermaid had put the open ends of the double blankets at the foot, instead of at the head, of the bed. I tried in vain to turn and right them. They were in coils and twists innumerable, and would not be righted. Finally, I slept, from sheer exhaustion; and this horrible cold that annoys me so much, is the result. But that night I swore a solemn oath, never, so long as I live, to go to bed again without having first examined the bed, to see if the blankets are put on right.' The lives of the most of us are made up of trifles, that vitally affect our happiness; and the wretched experience of my travelling acquaintance, the Professor, while showing the results of a seemingly trivial matter, forcibly illustrates the importance of knowing how to properly do so small a thing as put blankets on a bed!"

"Indeed, Kate," said Emmeline, "I think beds generally, outside of first-class hotels, are an abomination. People spend about one third of their lives in bed; and what should be a couch of rest, where sleep comes unbidden, is more often a rack of torture,—a humpy, bumpy, sliding, rolling, tormenting thing, so badly made up that you roll and toss, the night through, seeking vainly either rest or sleep. And the sickening odors of "spare rooms," or "guest-chambers," as they are termed, where weary visitors are put to spend the night on such beds! Bah! they linger in one's memory for years. You will do a good thing in your school, if you impress upon your pupils the necessity of comfortable, well-made beds, and thoroughly aired spare-rooms and parlors."

"Emmeline, our pupils will be taught to live in their homes—to occupy their parlors and spare rooms every day; and to keep them swept and garnished for themselves, and not for occasional guests. Self-respect is one of the first things that should be developed in a boy or girl. And by self-respect I mean the high and honorable feeling that prevents men and women from being mean and narrow to themselves in their daily home life. People who have genuine respect and consideration for themselves, never fail to treat other people with respect and consideration. To learn to be just and respectful to our fellow men and women, we must first learn to be just and respectful to ourselves. I have but little faith in reformers who go about endeavoring to reform society, while they neglect or are ignorant of the duties they owe to themselves and their families. Think you women who consider household work menial and degrading, and whose houses are nurseries of disorder and ill-training, are likely to elevate and advance humanity by their efforts? The true reformer must begin at home, and work outward. And until the average home is reformed and made what it should be, the reformatory movements of our age will mostly end in failure. Here among my letters is one from Mrs. Wheaton, a woman of wealth and culture, who feels that *she* was created for something better than to be 'a household drudge.' She asks, 'Can you suggest any way whereby the wife and mother can make homes happy and attractive to husband and sons, without giving her life to the work?—devoting all her powers of mind and body to it? In short, without bringing her mind down to the level of a common cook and drudge?' Now, the idea that certain kinds of labor are degrading, while other kinds are refining and elevating, is, I think, a stumbling block to many. People of education and intelligence seem to lose sight of the fact that it is the heart we put into labor of any kind—the motive that underlies it—that makes its performance either elevating or degrading in its effects. It seems strange to me that people who profess to believe in the dignity of labor, should make such absurd distinctions. And I am unable to see how the ordinary labor of the farmer, merchant, doctor, or lawyer, can be any more pleasant, interesting, or refining to them, than ordinary housework is to the wife and mother. Is there not as wide a field for the use of brains in her department as in each or any of their departments? And is it a small matter—a low ambition—to devote one's time and energies to making a pleasant, attractive home? Do not thousands of men toil uncomplainingly, day after day, all their lives, to give their wives and daughters the means to render home comfortable and attractive? For my part, I can conceive of no nobler aim in life for any woman than that of making a perfect home. Can she 'devote all her powers of mind and body' to any higher or holier object? I know of none. Hence my desire to aid in establishing this home-school, where girls can be taught to perform thoroughly all life's homely household duties."

"Kate, good men and women everywhere should lend a helping hand to your enterprise," said Emmeline, earnestly, after listening attentively to my sermonizing. "The subject assumes a new aspect to me; and I begin to realize that the wives and mothers who allow their daughters to grow up without being educated in all the branches of housekeeping, or who spend their lives in boarding-houses for the purpose of escaping domestic duties, are among the worst foes of society."

"Emmeline, your language is forcible, but just," was my reply. "Boarding-houses may be necessary; but they are necessary evils. They destroy all domestic privacy, and are demoralizing society by eating out the heart of our home life. Girls whose training in household duties has been neglected, flee after marriage to these places of refuge, to escape the penalty of their bad or neglected household training, and the oppression of ignorant, insolent servants; and thus boarding-houses perpetuate the system of which they are the legitimate result,—that wretched system under which women are reared without a knowledge of housework, and are taught to shirk the cares and responsibilities—thereby losing all the joys and comforts—of a Home!"

"But this school you speak of," interrupted Emmeline, "is not to be solely a training-school for girls in these mere domestic matters,—is it, Kate?"

"By no means," I answered. "The aim of its projectors is to make it a model school for girls, where they may enter at an early age, and continue as long as they please. I have no expectation that any of our girls will finish their education at school. They will be taught that education is a life-work,—that self-development and self-restraint are the primary objects of all education; and that until they have perfectly developed all their faculties, and are capable of restraining all their appetites and passions, their education will be incomplete. Instruction will be given in all the branches usually taught in a first-class school for young women, and in many more, as well as in domestic or household economy. In short, Cousin Emmeline, the object is to establish a home-school worthy of the name, worthy of the nineteenth century, and worthy of the women of America."

"Kate, notwithstanding the slur of the *Democrat*, you know I've never had any affiliation with 'strong-minded women.' On the contrary, I have been decidedly opposed to most of their movements; but I like this school project of yours. It seems to me a great deal better for women to go to work to correct our social evils in some such practical way than to go about clamoring for the ballot, and scolding men generally for depriving them of their rights. Have you ever talked with Alice about it? Perhaps it would strike her favorably. And if it should —"

"Why not go into it heart and soul yourself, Cousin Emmeline," I interrupted eagerly. "Why not put your life and fortune into it? Believe me, it would pay a great deal better than writing novels."

"Pay?" answered Emmeline, interrogatively. "I never thought of making money out of my novels."

"No, no, Emmeline; you misunderstand me. I think it will pay better than writing novels, in other ways than in money. It will pay in something better and more enduring than dollars and cents. I think it will pay in —"

"But," interrupted Emmeline, "the sun is setting—how beautiful!—and Alice will be wondering what has become of us. Let us drop the subject for the present, and go home."

---

Books, even more than other surroundings, carve their own features upon the minds and hearts of the young and impressible, and too much care and judgment can not be exercised in the selection of the literature of the school and family.

